# Immunosuppressive therapy versus supportive care in IgA nephropathy patients with stage 3 and 4 chronic kidney disease

**DOI:** 10.1097/MD.0000000000030422

**Published:** 2022-09-09

**Authors:** Gabriel Ștefan, Simona Stancu, Adrian Zugravu, Nicoleta Petre, Silviu Secăreanu, Otilia Popa, Cristina Capusa

**Affiliations:** a University of Medicine and Pharmacy Carol Davila, Bucharest, Romania; b Dr. Carol Davila Teaching Hospital of Nephrology, Bucharest, Romania.

**Keywords:** CKD stage 3 and 4, IgA nephropathy, immunosuppressive therapy, supportive care

## Abstract

The use of immunosuppressive therapy for immunoglobulin A nephropathy (IgAN) patients with stage 3 or 4 chronic kidney disease (CKD) is controversial. We performed a monocentric retrospective study on 83 consecutive IgAN patients with stage 3 or 4 CKD and proteinuria ≥0.75 g/d (age 41 [33–56] years, 72% male, estimated glomerular filtration rate 36.1 [25.4–47.5] mL/min/1.73 m^2^) who received uncontrolled supportive care (Supp) (n = 36), corticosteroids/corticotherapy (CS) (n = 14), or CS combined with monthly pulses of cyclophosphamide (CS + CFM) (n = 33) between 2010 and 2017. Patients were followed until composite endpoint (doubling of serum creatinine, end-stage kidney disease (dialysis or kidney transplant) or death, whichever came first) or end of study (January 2020). Patients were followed for a median of 29 (95% confidence interval = 25.2–32.7) months, and 12 (15%) patients experienced the composite endpoint. Within the limitation of a retrospective study, our results suggest no benefit from immunosuppressive therapy in patients with IgAN with stage 3 and 4 CKD as compared with supportive care.

There were no differences between the 3 studied groups regarding age, estimated glomerular filtration rate, proteinuria, Oxford classification score, arterial hypertension, and therapy with renin-angiotensin system inhibitors.

Mean kidney survival time for the entire cohort was 81.0 (95% confidence interval = 73.1–89.0) months, without significant differences between the 3 groups.

In univariate and multivariate Cox regression analysis adjusted for IgAN progression factors, immunosuppressive therapy was not associated with better kidney survival when compared with supportive therapy.

## 1. Introduction

Immunoglobulin A nephropathy (IgAN) is the most prevalent cause of primary glomerular disease in the world and one of the most frequent reasons for younger adults to require kidney replacement therapy.^[1,2]^

However, a considerable number of patients with IgAN is referred to a nephrologist only in advanced stages of chronic kidney disease (CKD) with estimated glomerular filtration rate (eGFR) 20 to 50 mL/min/1.73 m2 after a relatively silent but slowly progressive course.^[1]^ These patients are a conundrum from a therapeutic point of view since limited data from observational and randomized controlled trials are available.

Observational studies have suggested that corticotherapy might be of benefit when the eGFR drops under 50 mL/min/1.73 m^2^, especially in the presence of high level of proteinuria, but the threshold at which the treatment efficiency is lost is not well defined.^[3]^ However, subanalyses of the most recent randomized controlled trials failed to show any benefit of immunosuppressive therapy in patients with eGFR 30 to 60 mL/min/1.73 m^2^ in the STOP-IgAN and 20 to 50 mL/min/1.73 m^2^ in the Therapeutic Evaluation of Steroids in IgA Nephropathy Global (TESTING) trial.^[4–6]^ Moreover, a consensus, termed *the point of no return*, has emerged for patients with eGFR of 20 to 30 mL/min/1.73 m^2^ or less for whom immunosuppressive therapy is ineffective.^[7,8]^

Despite the higher number of clinical trials which investigated the benefits of various therapy regimens in IgAN during the last decade, there are still controversies due to inconsistent results especially for patients with moderate to severe kidney function decline, which were only scarcely enrolled. Therefore, in the present study, we aimed to evaluate the efficacy of 3 different treatment strategies in IgAN patients with eGFR from 15 to 59 mL/min/1.73 m^2^ and proteinuria higher than 0.75 g/d: supportive care, corticosteroid therapy, and corticosteroid therapy plus cyclophosphamide.

## 2. Methods

### 2.1. Patient selection

We performed a unicentric, retrospective, observational study on all patients with histologically proved IgAN from January 2010 to December 2017 at a university-affiliated tertiary-care referral center of Nephrology.

The inclusion criteria were primary IgAN with a stable eGFR from 15 to 59 mL/min/1.73 m^2^ (i.e., with variations of <30% in the 3 months before diagnosis) and proteinuria ≥0.75 g/d.

**Figure 1.**
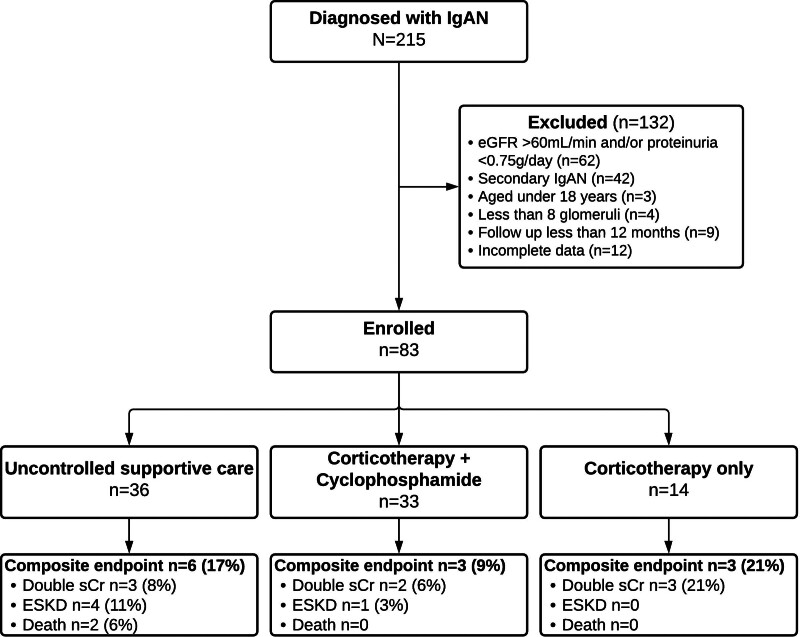
Patients’ flow chart. Composite endpoint: doubling of serum creatinine, ESKD (dialysis or kidney transplant) or death, whichever came first. eGFR = estimated glomerular filtration rate, ESKD = end-stage kidney disease, IgAN = immunoglobulin A nephropathy, sCr = serum creatinine.

Those with ages under 18, those whose kidney biopsy specimen contained <8 scorable glomeruli, with insufficient clinical data or with a shorter than 12 months follow-up at January 1, 2020 were excluded from the analysis, leaving a final cohort of 83 patients (Fig. 1).

Study patients were divided into 3 groups according to the treatment they received: uncontrolled supportive care (n = 36), corticosteroids/corticotherapy (CS) (n = 14), or CS combined with monthly pulses of cyclophosphamide (CS + CFM) (n = 33).

### 2.2. Data collection

Table 1 includes demographic data at kidney biopsy, histological findings, treatment, and outcomes. Data were retrieved from the electronic medical records of the enrolled patients.

**Table 1:** Baseline characteristics and outcomes in IgA Nephropathy patients.

	All N = 83	Supportive care n = 36	Immunosuppression			
			All n = 47	CS + CFM n = 33	CS only n = 14	*P* *
Age (y)	41 [33–56]	46 [33.5–61.0]	40 [33–47]	41 [34–48]	35.5 [32.0–42.0]	.2
Male gender (%)	72	67	77	82	64	.2
Charlson comorbidity index	2 [1–3]	2 [2–4]	2 [0–3]	2 [1–3]	2 [0–2]	.1
Obesity (%)	40	39	40	49	21	.2
Diabetes mellitus (%)	10	14	6	9	0	.3
Arterial hypertension (%)	93	94	92	94	86	.5
Serum creatinine (mg/dL)	2.1 [1.6–2.6]	1.8 [1.4–2.3]	2.1 [1.7–2.6]	2.4 [1.8–2.9]	1.8 [1.5–2.1]	.07
eGFR (mL/min/1.73 m^2^)	36.1 [25.4–47.5]	37.7 [27.5–49.2]	33.6 [25.5–46.0]	31.5 [22.7–44.3]	40.3 [32.5–54.6]	.08
Proteinuria (PCR, g/g)	1.7 [1.2–3.1]	1.9 [1.4–3.5]	1.5 [1.1–2.6]	1.7 [1.1–2.9]	1.3 [1.0–1.7]	.09
Hematuria (cells/mm^3^)	110 [30–230]	40 [29–195]	185 [30–250]	170 [30–240]	225 [98–350]	.03
Cholesterol (mg/dL)	217 [177–250]	223 [185–269]	202 [174–240]	200 [172–235]	221 [177–259]	.2
Triglycerides (mg/dL)	185.5 [121.0–248.0]	183 [128–252]	193 [119–245]	196 [118–241]	188 [121–318]	.8
Serum albumin (g/dL)	4.1 [3.8–4.4]	4.1 [3.8–4.4]	4.1 [3.8–4.4]	4.2 [3.8–4.5]	4.0 [3.8–4.4]	.8
Serum uric acid (mg/dL)	7.4 [6.0–8.4]	6.6 [5.0–7.8]	7.8 [6.5–8.6]	7.6 [6.6–8.6]	8.0 [6.3–8.6]	.06
Kidney biopsy						
M1 (%)	67	65	68	64	61	.1
E1 (%)	24	28	21	24	14	.6
S1 (%)	60	58	62	66	50	.5
T1/2 (%)	28/12	25/22	30/4	36/6	14/0	.04
C1/2 (%)	16/5	6/3	23/6	24/6	6/3	.2
MESTC score	3 [2–3]	2.5 [1.5–4.0]	3 [2–3]	3 [2–3]	2 [0–2]	.3
Treatment (%)						
RASI	74	83	66	67	64	.2
Outcome, n (%)						
Double sCr	8 (10)	3 (8)	5 (11)	2 (6)	3 (21)	.3
ESKD	5 (6)	4 (11)	1 (4)	1 (3)	0 (0)	.2
Death	2 (2)	2 (6)	0 (0)	0 (0)	0 (0)	.2
Composite endpoint (double sCr, ESKD, death)†	12 (15)	6 (17)	6 (13)	3 (9)	3 (21)	.4

C1/2 = crescents in at least 1 glomerulus, E1 = endocapillary hypercellularity, eGFR = estimated glomerular filtration rate (estimated with CKD-EPI formula), ESKD = end-stage kidney disease, IgAN = immunoglobulin A nephropathy, M1 = mesangial hypercellularity, PCR = urinary protein-to-creatinine ratio, RASI = rennin-angiotensin system inhibitor S1, segmental glomerulosclerosis, sCr = serum creatinine, T1/2 = tubular atrophy and interstitial fibrosis >25%.

* Comparison between supportive care versus CS + CFM versus CS only.

† Composite endpoint—doubling of serum creatinine, ESKD (dialysis or kidney transplant) or death, whichever came first.

The original kidney biopsies from all patients were available and were reviewed by an experienced nephropathologist. The diagnosis of IgAN was based on light microscopy, immunofluorescence (dominant IgA in the mesangium), and electron-microscopy (para-mesangial electron-dense deposits). All kidney biopsies were reviewed and scored according to the 2016 revised Oxford Classification (MESTC score): mesangial hypercellularity (M0/M1), endocapillary hypercellularity (E0/E1), segmental glomerulosclerosis (S0/S1), tubular atrophy/interstitial fibrosis (T0/T1/T2), and cellular or fibrocellular crescents (C0/C1/C2).^[9]^

### 2.3. Treatment

The corticosteroid only group consisted of patients who received the previously described Pozzi or Mano therapeutic regimes.^[10,11]^

The patients in the corticosteroid and cyclophosphamide group received monthly intravenous CFM pulse therapy (750 mg/m^2^ body surface area) over a period of 6 months and prednisone 40 mg/d (reduced to 5 mg/d by 6 months), then azathioprine 1.5 mg/kg/d continued for a minimum of 2 years.^[12–14]^

Intravenous CFM pulse regime was used because pulse therapy was shown to be safer due to short-term acrolein bladder exposure, and similarly efficient to oral CFM or other immunosuppressants in IgAN and other autoimmune diseases.^[12,13,15–17]^

The uncontrolled supportive care group consisted of patients who were not on any immunosuppressant.

Therapy with angiotensin converting enzyme (ACE) inhibitor or angiotensin receptor blocker were the first-line antihypertensives recommended for blood pressure control (targeting a sitting systolic blood pressure in 120 seconds). However, the indication and choice of treatment was at discretion of the nephrologist in charge of the patient. Therefore, a quarter of the studied patients were not on rennin-angiotensin system inhibitor therapy (Table 1).

### 2.4. Study endpoints

Patients were followed until composite endpoint (doubling of serum creatinine, ESKD [dialysis or kidney transplant] or death, whichever came first) or end of study (January 2020). All the included patients were evaluated regularly by the nephrologist in charge at least every 3 months in accordance with the local protocol for monitoring CKD stage 3 and 4.

### 2.5. Statistical analysis

Descriptive statistics were summarized as mean ± standard deviations or median with interquartile ranges (IQR) for continuous variables, as appropriate, and frequency distribution was presented with percentages for categorical variables. Differences between groups were assessed in case of continuous variables by Student *t* test or by Mann–Whitney and Kruskal–Wallis tests, according to their distribution, and in case of categorical variables by Pearson χ^2^ test.

The cumulative probability of event-free survival was assessed by Kaplan–Meyer method and the log-rank test was used for comparisons.

Univariate and multivariate Cox proportional hazard ratio (HR) analyses were performed to identify independent predictors of the composite endpoint. The results of Cox analyses are expressed as a HR and 95% confidence interval (95% CI); HR expresses the risk of the combined event (i.e., composite endpoint).

There were 2 treatment strategies studied with supportive care being the reference category: supportive care versus immunosuppressive therapy (CS + CFM and CS only) and supportive care versus CS + CFM versus CS only. Variables (i.e., treatment strategies) were entered into multivariate Cox regression models using an enter method, and the presented variables (Table 2) were derived from adjusted models: model 1 was adjusted for age, sex, hypertension, proteinuria, eGFR; model 2 was adjusted for the variables in model 1 plus the use of renin-angiotensin system inhibitors; model 3 was adjusted for the variables in model 2 plus MESTC score.

**Table 2:** Effects of immunosuppressive therapy on kidney survival (composite endpoint) in IgAN patients—Cox regression analysis.

	Immunosuppressive therapy		Supportive care	CS + CFM		CS only	
	HR (95% CI)	*P*		HR (95% CI)	*P*	HR (95% CI)	*P*
Univariate	1.34 (0.43–4.19)	.6	Ref.	0.51 (0.12–2.05)	.3	1.34 (0.33–5.40)	.6
Multivariate model 1*	1.45 (0.44–4.71)	.5	Ref.	0.25 (0.05–1.22)	.08	1.01 (0.24–4.26)	.9
Multivariate model 2†	2.10 (0.56–7.81)	.2	Ref.	0.16 (0.03–1.32)	.09	0.78 (0.16–3.64)	.7
Multivariate model 3‡	2.17 (0.54–8.64)	.2	Ref.	0.18 (0.02–1.20)	.07	0.85 (0.17–4.19)	.8

There were 2 treatment strategies studied with supportive care being the reference category: supportive care versus immunosuppressive therapy (CS + CFM and CS only) and supportive care versus CS + CFM versus CS only.

CFM = cyclophosphamide, CI = confidence interval, CS = corticosteroids/corticotherapy, eGFR = estimated glomerular filtration rate, HR = hazard ratio, MESTC = Oxford classification, RASI = renin-angiotensin system inhibitor, ref. = reference.

* Model 1 was adjusted for age, sex, hypertension, proteinuria, eGFR.

† Model 2 was adjusted for age, sex, hypertension, proteinuria, eGFR, RASI.

‡ Model 3 was adjusted for age, sex, hypertension, proteinuria, eGFR, RASI, MESTC score.

In all analyses, *P* values are 2-tailed and all *P* values <.05 were considered statistically significant.

Statistical analyses were performed using the SPSS program (SPSS version 20, Chicago, IL).

### 2.6. Ethics

The study was conducted with the provisions of the Declaration of Helsinki and the protocol was approved by the local ethics committee (The Ethics Council of “Dr Carol Davila” Teaching Hospital of Nephrology, registration number 21/June 30, 2020).

The need for informed consent was waived due to exclusive use of deidentified information and the retrospective nature of the study.

## 3. Results

### 3.1. Study population

The study population included 83 patients (72% male). At the time of IgAN diagnosis, their age was 41 (IQR 33–56) years, eGFR was 36.1 (IQR 25.4–47.5) mL/min/1.73 m^2^, proteinuria was 1.7 (IQR 1.2–3.1) g/g and most patients had arterial hypertension (93%).

The median comorbidity index evaluated with Charlson score was 2.0 (IQR 0–3) (Table 1).

Histopathological assessment revealed that mesangial hypercellularity was present in 67% of patients, endocapillary hypercellularity in 24%, segmental glomerulosclerosis in 60%, tubular atrophy and interstitial fibrosis >25% (T1 + T2) in 40% and 21% showed crescents in at least 1 glomerulus. The median MESTC score was 3 (IQR 2–3) (Table 1).

More than half of the patients received immunosuppression therapy (47, 57%), while the rest were on uncontrolled supportive care (36, 43%). The immunosuppressive therapy group was further categorized into the CS only group (14, 17%) and the CS + CFM group (33, 40%) (Table 1).

Patients were followed for a median of 29 (95% CI = 25.2–32.7) months, and 12 (15%) patients experienced the composite endpoint (Table 1).

### 3.2. Comparison between the treatment groups

There were no differences between the 3 studied groups regarding age, eGFR, proteinuria, MESTC score, hypertension, and therapy with renin-angiotensin system inhibitors (Table 1).

Mean kidney survival time for the entire cohort was 81.0 (95% CI = 73.1–89.0) months.

**Figure 2.**
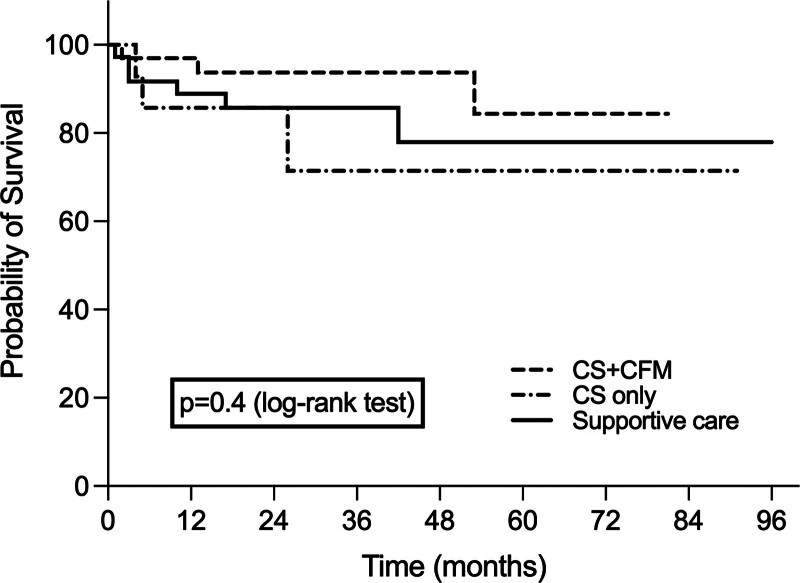
Cumulative event-free survival (Kaplan–Meier analysis), there was similar kidney survival time between the 3 groups. CS = corticosteroids/corticotherapy, CFM = cyclophosphamide.

We found similar kidney survival time between the 3 groups (supportive care 79.0 [95% CI = 66.5–91.6] vs CS 69.3 [95% CI = 47.7–91.0] vs CS + CFM 73.7 [95% CI = 66.0–81.4] months, *P* = .4; Fig. 2).

In univariate and multivariate Cox regression analysis adjusted for IgAN progression factors, immunosuppressive therapy was not associated with better kidney survival when compared to supportive therapy (Table 2).

### 3.3. Adverse events

There were no significant differences in the number of fatal events between the 2 groups or between the 2 subgroups in the immunosuppression arm (Table 1).

The incidence of other adverse events was also similar between the 3 studied groups. Thus, there were no differences in the number of hospitalizations required for infection (2 in the supportive care group, 3 in the CS + CFM group, and 2 in the CS only group; *P* = .7). Only 8 patients experienced leukopenia during the study period (2 in the supportive care group, 3 in the CS + CFM group, and 3 in the CS only group; *P* = .8). Also, diabetes mellitus related to corticotherapy was not reported during the follow-up.

## 4. Discussion

Considering the diverse spectrum of clinical course in IgAN, our study focuses on patients with moderately advanced CKD for whom the use of immunosuppressive therapy is controversial. We found that patients with IgAN with stage 3 and 4 CKD who received immunosuppression had similar kidney survival when compared to those only on supportive therapy.

More than 20 years ago, a *point of no return* was suggested: for serum creatinine values higher than 3 mg/dL, IgAN inexorably progresses toward ESKD.^[7]^ This limit of *no return* has been confirmed by other investigators in observational studies and further reduced to 2 mg/dL.^[8,18]^ However, most of the performed randomized controlled trials excluded IgAN patients with eGFR under 30 mL/min/1.73 m^2^ and enrolled few patients with eGFR between 30 and 60 mL/min/1.73 m^2^. Our study population encompasses these patients, since the median eGFR at diagnosis was 36.1 mL/min/1.73 m^2^ indicating advanced kidney function impairment. Furthermore, chronic lesions like glomerulosclerosis and tubular atrophy with interstitial fibrosis were present in more than half of our cohort.

There is compelling evidence that an optimized supportive treatment is the mainstay of IgAN management.^[19]^ Lowering blood pressure to a target of systolic BP in 120 seconds has been shown to reduce CKD progression in IgAN.^[19]^ Reaching a mean blood pressure of 129/70 mm Hg prevented the decline in kidney function over 3 years which was observed in patients achieving a mean blood pressure of 136/76 mm Hg.^[20]^ Moreover, 2 prospective, controlled studies support the use of ACE inhibitors as first-line antihypertensive drugs in IgAN patients.^[21,22]^

The risk for progression in IgAN decreases significantly if proteinuria can be reduced to under 1 g/d.^[23]^ ACE inhibitors have superior antiproteinuric effect as compared with other hypotensive agents.^[21,22]^

Most of the patients in the supportive arm of our study and approximately two-thirds of the patients receiving immunosuppression were on a renin-angiotensin system inhibitor. Importantly, there were no differences at diagnosis between the studied groups regarding the recognized risk factors for CKD progression: GFR, proteinuria, hypertension, MESTC score.

Currently, high risk of progression in IgAN is defined as proteinuria higher than 0.75 to 1 g/d despite at least 3 months of optimized supportive care. These patients are considered for a 6-month course of corticotherapy. However, even in these high-risk groups, the efficacy of corticotherapy and other immunosuppressive agents is still a controversial topic.

In a retrospective study of the validate the Oxford Classification of IgAN in a European cohort cohort which used propensity score matching, corticosteroid therapy slowed the rate of kidney function decline and increased the survival without ESKD or a 50% decline in kidney function.^[3]^ Moreover, the beneficial effect was more evident in patients with eGFR < 50 mL/min/1.73 m^2^ and with higher levels of proteinuria.^[3]^

In a similar study design with ours, Ma et al^[24]^ reviewed 132 consecutive IgAN patients with stage 3 or 4 CKD and proteinuria ≥1.0 g/d who received uncontrolled supportive care (n = 41), corticotherapy (n = 22), or low dose corticosteroids combined with oral cyclophosphamide (n = 69). The combined endpoint was defined as either a ≥50% reduction in eGFR or ESKD.^[24]^ They found that low dose corticotherapy combined with oral cyclophosphamide is more effective than uncontrolled supportive care for IgAN patients with reduced kidney function.^[24]^

In the STOP-IgAN trial, 162 patients with proteinuria >0.75 g/d and eGFR > 30 mL/min/1.73 m^2^ were randomized—after 6 months of optimal supportive care—to supportive treatment versus 2 immunosuppression regimens (corticotherapy if eGFR > 60 mL/min/1.73 m^2^ and corticotherapy plus cyclophosphamide/azathioprine if eGFR < 60 mL/min/1.73 m^2^).^4]^ Even though the number of patients who reached remission was significantly higher in the immunosuppression arm (18% vs 5%), there were no differences in the loss of kidney function (i.e., ≥15 mL/min/1.73 m^2^).^4]^ Moreover, in a 10-year follow-up study of 149 of 162 patients randomized in the STOP-IgAN trial, Rauen et al^[5]^ report that there were no differences in outcome between the supportive care only group and the supportive care plus immunosuppression group. Interestingly, patients with eGFR < 60 mL/min/1.73 m^2^ who received cyclophosphamide-azathioprine experienced a tendency to poorer outcomes at between 2 and 6 years but not at the end of follow-up as compared to the supportive care only subjects.^[5]^

The TESTING study was a randomized, double-blind, controlled study which aimed to compare corticosteroid treatment with placebo in IgAN patients with eGFR between 20 and 120 mL/min/1.73 m^2^ and proteinuria >1 g/d.^[6]^ Despite favorable effects on proteinuria and eGFR decline, the risk of adverse events was higher in the treatment arm, so it was suspended early after randomizing only 262 of the more than 700 initially planned patients.^[6]^ However, a subgroup analysis did not show the effects of corticotherapy on the composite outcome in the patients with eGFR < 50 mL/min/1.73 m^2^.^[6]^

Our results are in line with the subanalysis performed in the randomized control trials, suggesting that therapeutic management of IgAN patients with CKD 3 and 4 should be based on non-immunosuppressive measures, collectively known as supportive care. This is further supported since recent studies with systemic immunosuppression in IgAN have also raised safety concerns.

Frequently, IgAN patients present at diagnosis with moderately advanced CKD, that is, CKD stage 3 and 4, after a silent but slowly progressive course and ESKD occurs in up to 50 percent of the patients after 20 to 25 years.^[21]^ In these patients, angiotensin II inhibition with an ACE-inhibitor or angiotensin receptor blocker slows the rate of progression of most proteinuric CKD, an effect that is mediated at least in part by lowering both the systemic blood pressure and the intraglomerular pressure, thereby reducing both proteinuria and secondary glomerular injury.^[21,22,25]^

There were some limitations to the present study. First, we performed a single center retrospective observational study on a relatively small number of patients, and it is difficult to control for all factors that may affect kidney survival. Second, selection bias between patients who received immunosuppression and those who received uncontrolled supportive care was inevitable, since the therapeutic regimens were according to the physician experience. The retrospective design also limited the analysis regarding the adverse events, and we could only include major adverse events that were registered in the hospital electronic records.

Within the limitation of a retrospective study, we found no benefit from immunosuppressive therapy in patients with IgAN with stage 3 and 4 CKD as compared with supportive care. However, ethnically diverse randomized controlled studies with long-term follow-up focused on retarding CKD progression are needed in these patients.

## Author contributions

Conceptualization: G.S., S.S., and C.C.; methodology: G.S., S.S; validation: C.C., A.Z., and S.S.; formal analysis: G.S.; investigation: S.S., N.P., and O.P; data curation: A.Z., S.S., N.P., and O.P; writing—original draft preparation: G.S., S.S.; writing—review and editing: C.C., S.S.; supervision: C.C.; all authors have read and agreed to the published version of the manuscript.
